# A Treatise on Diseases of the Genito-Urinary Organs

**Published:** 1874-09

**Authors:** 


*A Practical Treatise on Diseases of the Genito-Urinary Organs, including Syphilis. Designed as a Manual for Students and Practitioners. With Engravings and Cases.* By W. H. VAN BUREN, A. M., M. D., etc., and E. L. KEYES, A. M., M. D., etc. New York: D. Appleton & Co., 1874. Buffalo: Herger & Ulbrich.

This work has been promised to the profession for some time and has been looked forward to as something which would take a high position in the medical literature of the day. We are told in the preface that "its object is to present to the student and general practitioner a succinct account of the nature and treatment of the diseases incident to the genito-urinary organs as they are encountered in private, and hospital practice, by those engaged in their daily and especial study. The literature of this department of surgery has been exhaustively studied with the purpose of reproducing every fact of practical value."

The student of the work will, we think, find that the authors have accomplished their design in a large measure.

The first part embraces diseases of the genito-urinary organs. Chapters I, to VII, are devoted to diseases of the penis; chapter first being devoted to the anatomy and diseases of the penis in general, and chapters two to seven embracing diseases of the urethra, as gonorrhœa, stricture, etc. These subjects are well treated, and this section is calculated to be an excellent guide in the treatment of diseases of these parts. Chapters X and XI are occupied with a consideration of disease of prostate, and chapters XII and XIII with affections of the bladder, not including stone. Chapter XIV discusses stone in the bladder, and the remaining four chapters are devoted to lithotomy and lithotrity. These subjects are exceedingly well discussed, although not to that extent which would be desired in a work of larger size.

Chapter XIX upon diseases of the ureters, occupies less than a page. Chapter XX is upon diseases of the kindneys, and chapters XXI to XXVIII close this section of the work in an account of the maladies of the scrotum, testicles, those involving the genital function, of the cord, and of the vas defferens and seminal vesicles.

Part II is upon Chancroid and Syphilis, and consists of thirteen chapters. The authors are positive in their statements regarding the distinctive features between chancroid and syphilis; regarding the result of inoculation with chancroidal virus as always local that from syphilitic constitutional; chapter VIII upon syphilitic diseases of the eye, and chapter IX upon syphilitic diseases of the ear, are from the pens of Profs. Noyes, and Roosa. Chapter XII upon syphititic diseases of the nervous system, is an admirable review of this subject, and is a repetition of the views advanced by Prof. Keyes in an article in the *New York Medical Journal* for November, 1870.

In the treatment of syphilis the authors advise the use of mercury during the primary stage, continuing it from six months to a year after all symptoms have ceased to manifest themselves. Iodide of Potassium, tonics &c., are recommended in the tertiary manifestations.

The whole subject of syphilis and chancroid is treated in accordance with the latest and best views upon the subject, and we are only sorry that the authors were so limited to the short space of thirteen chapters in its consideration.

The work is one of the most valuable contributions to medical science which has been published in some time. We congratulate Drs. Van Buren and Keyes upon their success.

